# Exploring the treatment of osteomyelitis based on nanozyme-based antibacterial strategies

**DOI:** 10.3389/fmedt.2026.1779701

**Published:** 2026-03-31

**Authors:** Junzhen Wu, Shuo Duan, Shuaiwei Li, Yisi Wang, Minglei Zhang

**Affiliations:** Department of Xinmin Orthopedic, China-Japan Union Hospital of Jilin University, Changchun, China

**Keywords:** antibacterial activity, catalytic therapy, nanozyme, synergistic therapy, treatment of osteomyelitis

## Abstract

Osteomyelitis is a severe bone infection caused by bacterial invasion, posing significant therapeutic challenges due to biofilm formation and antibiotic resistance. In recent years, nanozymes—a class of functional nanomaterials with enzyme-like catalytic activities—have shown considerable potential in antimicrobial applications owing to their high stability, tunable catalytic activity, and low tendency to induce resistance, positioning them as promising alternatives to conventional antibiotics. This review systematically examines the therapeutic prospects of nanozyme-based delivery systems for treating osteomyelitis. We detail the antibacterial mechanisms of nanozymes exhibiting various enzyme-mimicking activities—primarily peroxidase-, oxidase-, haloperoxidase-, and hydrolase-like functionalities—such as generating reactive oxygen species (ROS) by catalyzing endogenous hydrogen peroxide and physically degrading biofilm components. This review aims to provide a theoretical foundation and novel insights for developing efficient and safe nanozyme-based antimicrobial agents against osteomyelitis.

## 1 Introduction

Osteomyelitis refers to the invasion of bone tissue by bacteria, leading to bone destruction and erosion into surrounding tissues, with a high propensity for recurrence after treatment. Despite rapid advancements in medical technology, the prevalence of osteomyelitis continues to rise. Statistics indicate that the prevalence of osteomyelitis in the United States increased from 0.114‰ to 0.244‰ between 1979 and 2009 (1), in Spain from 0.0234‰ to 0.0578‰ between 1991 and 2011 (2), in Germany from 0.155‰ to 0.167‰ between 2008 and 2018 (3), and in South Korea from 0.078‰ to 0.091‰ between 2008 and 2016 (4).

The most common pathogens causing bone infections are staphylococci. *Staphylococcus aureus* (*S. aureus*) the most frequently identified pathogen, with bacterial cultures from approximately two-thirds of infected tissues revealing *S. aureus* or *coagulase-negative staphylococci* (5). Notably, about half of the osteomyelitis cases caused by *S. aureus* are attributable to methicillin-resistant *Staphylococcus aureus* (MRSA) strains (6). The pathophysiological mechanisms by which *S. aureus* destroys bone tissue include invasion of the osteocyte lacuno-canalicular network, intracellular infection, formation of staphylococcal abscess communities, and biofilm formation (7). The current primary treatment strategy for bone infections involves systemic antibiotic administration. However, the combined action of these mechanisms enables bacteria to evade host immune attacks and develop drug resistance. According to the European Centre for Disease Prevention and Control, antibiotic resistance increased by 65%–165% in some European countries between 2003 and 2012 (8). Concurrently, while bacterial antibiotic resistance was increasing, the number of new antibiotics approved annually by the US Food and Drug Administration decreased from 19 to just 1 between 1980 and 2012. Consequently, finding alternatives to antibiotics is necessary.

Researchers have turned their attention to drawing inspiration from enzymes possessing antimicrobial activity, which can cause irreversible damage to bacteria or disrupt biofilm integrity (9–13). Nanozymes, representing the state-of-the-art in artificial enzymes, are widely studied and applied across various fields due to their high catalytic activity, excellent stability, and low cost (14). Their extensive research in the antimicrobial field positions them as promising effective substitutes for antibiotics. Unlike conventional antibiotics, nanozymes are less likely to induce bacterial resistance, owing to the favorable membrane permeability and good biocompatibility of nanomaterials (15). Furthermore, due to their unique physicochemical properties, nanozymes can exhibit characteristics beyond those of natural enzymes, allowing for the modulation of their catalytic activity by altering their shape, size, and composition (16). These unique properties enable the development of multifunctional antimicrobial agents. Currently, antimicrobial nanozymes primarily include oxidoreductase mimics and hydrolase mimics (17).

However, the further implementation of nanozyme therapy for osteomyelitis remains significantly hindered because nanozymes cannot fundamentally distinguish between bacterial and mammalian cells, and their intrinsic catalytic performance is often insufficient, leading to suboptimal therapeutic efficiency (18). This review summarizes the antimicrobial mechanisms and latest advances of nanozymes with different enzyme-mimicking activities against bacteria and biofilms. Additionally, it summarizes methods and insights for conferring selectivity of nanozymes towards bacteria over mammalian cells. Finally, it highlights the key challenges and future perspectives in the research and application of antimicrobial nanozymes. By categorizing antimicrobial nanozymes based on their mimicked enzymatic activities, this review aims to provide researchers with new ideas and directions to overcome current obstacles in nanozyme development and application, ultimately facilitating the creation of effective antibiotic alternatives.

## 2 Mimicking oxidoreductases to combat osteomyelitis

Oxidoreductases play crucial roles in biochemical reactions by converting various substrates into different products, exhibiting excellent biological activity and high catalytic efficiency. Enzyme mimics possessing oxidoreductase-like activity primarily include peroxidase mimics, catalase mimics, superoxide dismutase mimics, oxidase mimics, and haloperoxidase mimics. Among these, peroxidase mimics, oxidase mimics, and haloperoxidase mimics demonstrate antimicrobial properties. They exert bactericidal effects by generating toxic ROS, which cause damage to *S. aureus*. Consequently, they are regarded as a new generation of antimicrobial agents and show significant potential for treating osteomyelitis.

### 2.1 Peroxidase mimics against osteomyelitis

Hydrogen peroxide (H₂O₂) has long been used in the antimicrobial field. It functions by inducing the oxidation of biomolecules, thereby impairing bacterial functions; however, this process is relatively slow. Thus, high concentrations of H₂O₂ are typically required to achieve the desired antibacterial effect, which can, unfortunately, damage healthy tissues and even delay wound healing. Peroxidase mimics simulate the activity of peroxidases, facilitating the conversion of H₂O₂ to water while oxidizing substrates into their corresponding oxides. Due to their high oxidative capacity, peroxidase mimics exhibit negligible bacterial resistance. The high levels of ROS generated can damage bacterial lipids, proteins, and the biofilm matrix. Nanozymes, including metal-based peroxidase mimics, carbon-based peroxidase mimics, and metal-organic framework (MOF)-based peroxidase mimics, have shown promising results in treating bacterial infections.

The inherent properties of metals enhance nanoscale materials and confer enzyme-mimicking characteristics. Common metallic nanomaterials such as Cu (19, 20), Fe (21, 22), and their oxides have been reported to function as peroxidase mimics, acting as bactericidal agents via Fenton or Fenton-like reactions (23, 24). The bactericidal efficacy of ·OH exceeds that of H₂O₂ exhibits lower reactivity and can be detoxified by endogenous antioxidants. In essence, benefiting from the involvement of copper or iron ions in catalyzing the conversion of H₂O₂ into hydroxyl radicals (•OH), the accelerated generation rate of •OH is closely correlated with significant antimicrobial activity.

**Figure 1:**
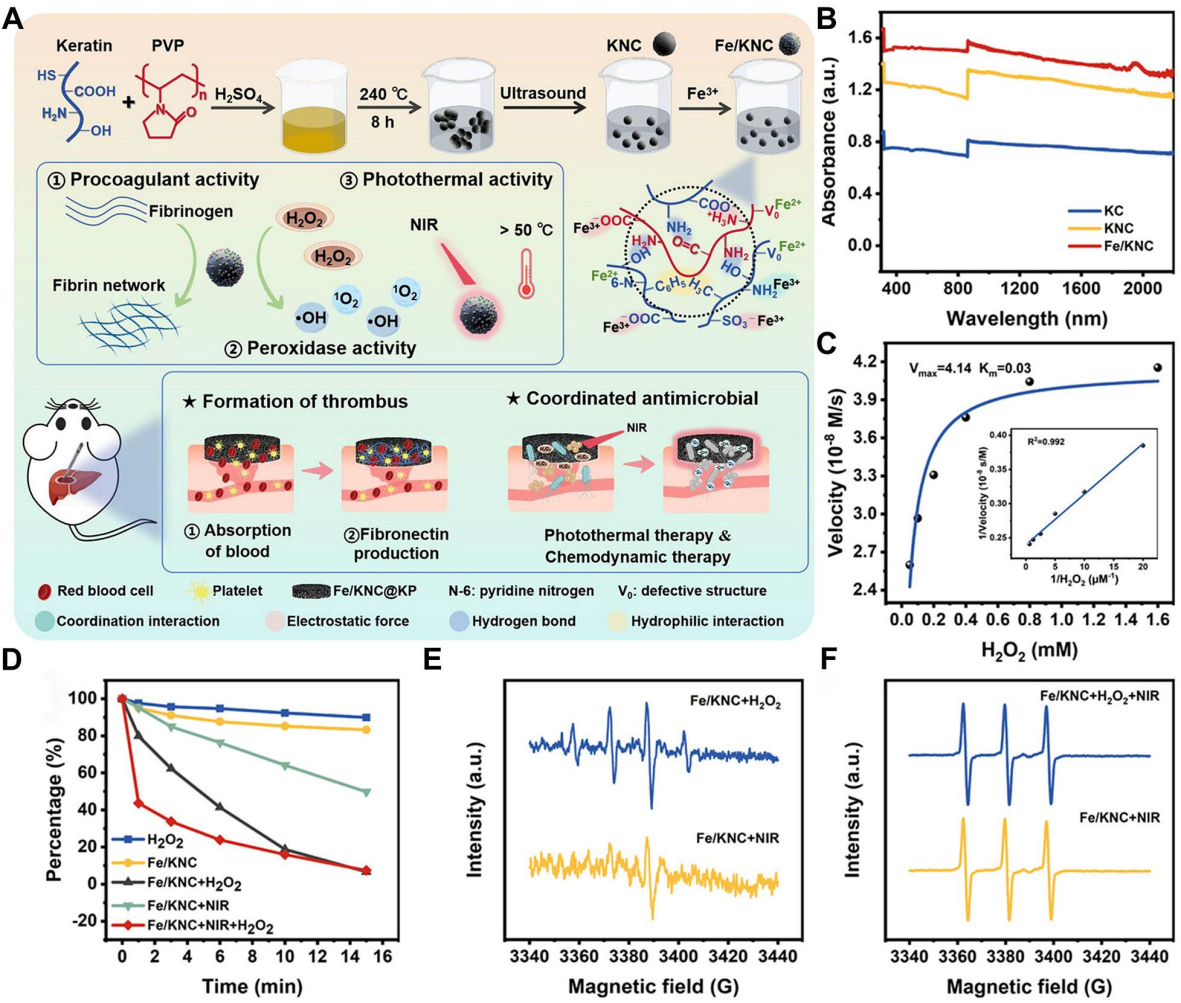
Antibacterial mechanism and effect of nanozymes (Fe/KNC). **(A)** Synthetic route of antimicrobial nanozymes. **(B)** UV–Vis-NIR of KC, KNC, and Fe/KNC. **(C)** Steady-state kinetic assay of Fe/KNC for different concentrations of HO. **(D)** Absorption spectra of the DPBF probes with different treatment. ESR spectra of different dispersions with **(E)** DMPO probes and **(F)** TEMP probes. All statistical data are presented as the mean ± standard deviation (SD; *n* = 3). Reproduced with permission (33).

Iron-based nanozymes utilized for antimicrobial purposes primarily include iron oxide nanozymes, Prussian blue nanozymes (PBNzymes), and iron sulfide nanozymes (ISNzymes). Among them, Fe₃O₄ nanozymes have garnered significant attention due to their effective peroxidase-like activity. These Fe₃O₄ nanozymes generate •OH radicals via iron-specific Fenton chemistry (Figure 1). However, their activity is pH-dependent, largely confined to acidic environments, with a substantial loss of antibacterial activity observed at near-neutral pH. Generally, most bacteria responsible for osteomyelitis (such as *S. aureus*, *Streptococcus pyogenes*, and *Escherichia coli*) actively proliferate at near-neutral pH values ranging from 6 to 8. Therefore, it is crucial to extend the peroxidase-like activity of Fe-based nanozymes into the neutral pH range. Vallabani et al. (25) addressed the pH dependency of Fe₃O₄ nanozymes by employing adenosine 5′-triphosphate disodium salt as a synergist. This approach accelerated the generation of •OH radicals and restored the antibacterial activity of the nanozymes across a broad pH spectrum. The pH limitation of Fe₃O₄ was effectively overcome by the synergistic effect provided by ATP (26).

PBNzymes represent a promising class of coordination polymers characterized by good biocompatibility, modifiable features, and magnetic properties, enabling broad applications in biomedicine. PBNzymes leverage their ability to penetrate the cell membrane of *S. aureus* and engage in selective ion exchange with intracellular iron, thereby disrupting iron metabolism within these pathogenic bacteria (27, 28). Wang et al. (27) reported a Prussian blue nanozyme, featuring a three-dimensional coordination polymer framework. This nanozyme demonstrated remarkable antibacterial activity by disrupting bacterial iron acquisition and metabolism. To investigate the cellular uptake, intracellular iron depletion, and antibacterial properties of PBNzymes, the authors utilized *S. aureus*—a common pathogen in osteomyelitis—as an *in vitro* bacterial cell model.

Iron sulfide participates in numerous cellular metabolic processes, electron transfer reactions, and the maintenance of enzymatic biological activity (29, 30). For instance, through microbially catalyzed formation of Fe-S protein clusters, it is possible to obtain metal clusters selective for *S. aureus* (31). Although ISNzymes are currently seldom applied in the antimicrobial field, their potential in this area should not be overlooked. ISNzymes act as effective catalysts that enhance the antimicrobial effect of H₂O₂. Wang et al. (32) discovered that under identical reaction conditions, ISNzymes exhibited an activity enhancement of nearly 652 times compared to IONzymes. This superior activity of ISNzymes can be attributed to the release of polysulfides, their higher specific surface area, and greater affinity for H₂O₂.

Zhang et al. prepared a multifunctional material based on nanozymes, which exhibited excellent peroxidase activity and showed significant antibacterial activity against *S. aureus* and Escherichia coli. It inhibited bacterial growth and biofilm formation by generating ROS (33).

In addition to the extensively researched iron-based nanozymes, copper-based nanozymes functioning as peroxidases have also garnered significant attention. In recent years, insufficient ROS generation and bacterial overexpression of glutathione have limited the efficacy of nanozymes against bacterial infections. Copper-based nanozymes exhibit effects similar to their iron-based counterparts. Cu^2+^ ions can efficiently catalyze the conversion of H₂O₂ into •OH, with a catalytic efficiency significantly higher than that of Fe^2+^. Furthermore, copper-based nanomaterials not only possess strong photothermal properties but also maintain their catalytic activity under extreme pH and temperature conditions (34, 35). Increasing temperature can accelerate chemical reaction rates. Therefore, elevating the temperature at the bacterial infection site to enhance the catalytic rate and antibacterial capacity of nanozymes represents a potential new direction for osteomyelitis treatment. However, for *in vivo* antibacterial applications, heating nanozymes via an external heat source is challenging to achieve. Consequently, leveraging the strong photothermal properties of copper-based nanozymes, near-infrared light irradiation combined with copper-based nanozymes presents a promising strategy (36).

Furthermore, copper-based nanozymes also demonstrate glutathione depletion capabilities, thereby enhancing the therapeutic efficacy of ROS-related treatments (37). Building on this, developing copper-based nanozymes with high catalytic performance and antimicrobial functions holds significant importance for the antibacterial treatment of osteomyelitis. Wang et al. (38) employed a pyrolysis-etching-adsorption-pyrolysis strategy to construct Cu single-atom sites/N-doped porous carbon for photothermal catalytic antibacterial treatment. The Cu SASs/NPC effectively induces peroxidase-like activity in the presence of H₂O₂, generating a substantial amount of •OH, which exerts a certain bactericidal effect and makes the bacteria more susceptible to temperature. The introduction of NIR can generate hyperthermia to combat bacteria and enhance the peroxidase-like catalytic activity, resulting in the production of additional •OH to eliminate bacteria. This copper-based nanozyme can also deplete glutathione within the bacteria, significantly improving the bactericidal effect. It demonstrated nearly 100% antibacterial efficiency against both *Escherichia coli* and methicillin-resistant *S. aureus*, showed excellent efficacy in treating infected wounds in mice, and shows promise as an effective therapy for MRSA-induced osteomyelitis.

Additionally, copper-based nanozymes exhibit valence-dependent peroxidase-like activity, where zero-valent copper and divalent copper (Cu^2+^) demonstrate distinct POD-like activities and antibacterial capabilities. Divalent copper (Cu^2+^) exhibits low POD-like activity, primarily exerting its antibacterial effect through a copper ion release mechanism. In contrast, zero-valent copper possesses high POD-like activity, with its antibacterial performance being predominantly determined by the ROS generated via a copper-catalyzed mechanism (39). These findings provide valuable direction for the design of novel antibacterial agents.

Gold-based nanozymes, characterized by their excellent photothermal conversion efficiency and high POD-like activity, hold promise for the clinical application of nanozyme therapy in osteomyelitis. For instance, Yan et al. (40) synthesized Au nanosheets using polyvinylpyrrolidone K132 as a template. Under low-power, short-duration laser irradiation, these nanosheets achieved a photothermal conversion efficiency of 68.5%, approaching the highest reported efficiency for gold nanosheets. Coupled with their high POD-like activity, they effectively eliminate bacteria while protecting normal tissues from damage caused by excessive irradiation, facilitating safe and rapid wound healing.

Furthermore, other metal-based nanozymes, including those based on Ag, Pt, and Pd, exhibit very similar antibacterial enzyme-like activities and pH dependence. Silver-based nanozymes rely on their high POD-like activity synergizing with the toxic effect of Ag^+^ ions on bacteria to promote the death of drug-resistant bacteria. However, due to the tendency of nanostructured Ag to aggregate in the bacterial microenvironment and the uncontrolled release of Ag^+^ ions, they often suffer from low bioavailability and high toxicity to organisms (41). The amphiphilicity of metal nanoclusters and their active nano-bio interactions make the use of silver-based nanozymes against pathogens more controllable (42, 43). Advanced silver nanoclusters are formed through precise control of their composition at the atomic level, enabling diverse modifications of the type and composition of surface ligands on silver-based nanozymes. This results in nanozymes with precise size, composition, and surface properties (44, 45). Chen et al. (46), by altering the size, core structure, and surface ligands, discovered that such silver-based nanozymes possess a remarkable capacity for •OH radical production, nearly twice the catalytic effect of AgNPs. These results essentially indicate that Ag nanoclusters exhibit significantly higher peroxidase-like activity compared to AgNPs (47).

As early as 2011, Shi et al. (48) discovered that carbon quantum dots (CQDs) can exhibit POD-like catalytic activity. Their unique optical and physicochemical properties make them ideal candidates for enzyme mimics. For instance, their nanocrystalline core and conjugated π-system endow them with excellent electron transfer capabilities (49). Furthermore, the abundant oxygen-containing functional groups on the surface of CQDs, such as −C = O, O = C-O-, and −O-H, can provide numerous favorable catalytic and substrate-binding sites for intrinsic peroxidase-like activity (50). However, the catalytic activity exhibited by CQDs is highly dependent on conditions such as pH and temperature. Current research has updated their catalytic properties through doping strategies. The synergistic effect of multiple heteroatom doping within the carbon lattice can instantly provide a high density of active sites and generate new characteristics to enhance catalytic activity (51). More importantly, the strong interaction between the heteroatom-doped CQD components and surface functional groups can also effectively improve their catalytic properties by accelerating intermolecular electron transfer (52).

For example, nitrogen doping can modulate the local electronic characteristics of CQDs, including charge delocalization between adjacent carbon atoms, thereby providing enhanced chemical reactivity (53). Phosphorus doping increases electron delocalization and introduces structural defects through the generation of new charge sites. Sulfur doping can modulate the density of excited states, enhancing the polarizability, structural defects, and spin density of CQDs (54, 55). Copper doping enables Fenton-like reactions, which can be utilized for reactive chemodynamic therapy. Copper-doped CDs have demonstrated excellent antibacterial activity even at doses as low as 5 μg/mL (56). Tripathi et al. (57) developed N, S, and P co-doped CQDs as nanozymes to mimic peroxidase activity. These NSP-CQDs exhibited catalytic activity across a broad pH range, including at neutral pH.

Graphene-based and graphene oxide-based nanozymes have been extensively investigated due to their remarkable bactericidal activity against a wide spectrum of bacteria. The antibacterial mechanisms of graphene-based nanozymes encompass both oxidative stress and membrane stress (58). Oxidative stress involves graphene oxide mimicking peroxidase-like activity to generate ROS that disrupt critical bacterial structures. Membrane stress refers to the physical damage inflicted by the sharp edges of nano-sized graphene oxide sheets, which can cut microbial cell walls and/or membranes. This damage leads to the leakage of cellular components into the environment, resulting in microbial death (59).

Graphitic carbon nitride (CN), a graphene-like metal-free polymer composed of triazine rings (60), has garnered significant attention from researchers as a non-toxic natural enzyme mimic due to its excellent physicochemical properties and biocompatibility (61, 62). CN exerts its antibacterial effect by activating H₂O₂ to generate reactive •OH. However, its limited peroxidase-like catalytic efficiency poses a bottleneck for its application in the antimicrobial field. Introducing high concentrations of nitrogen vacancies (NVs) into CN represents a promising strategy. The incorporation of NVs disrupts the s-triazine heterocycles, leading to alterations in local electron distribution, extended π-electron delocalization, and the exposure of numerous coordinatively unsaturated sites (63). These sites can not only adsorb substrates but also efficiently activate H₂O₂ to generate abundant ·OH intermediates, which is likely to enhance its antibacterial activity.

Therefore, Dai et al. (64) utilized an ultrasonic fragmentation method to break down bulk CN with a macroscopic structure into ultra-thin carbon nitride quantum dots with a uniform size of 4–5 nm, creating carbon nitride quantum dot nanozymes. In the presence of low concentrations of H₂O₂, these CNQDs demonstrated a sterilization rate exceeding 99% against *S. aureus*. Benefiting from the excellent antimicrobial properties of both CN and graphene oxide, Li et al. (65) prepared an outstanding artificial enzyme by combining hetero-lamellar g-C₃N₄ with graphene oxide via electrostatic bonding and π-π stacking interactions. This artificial enzyme achieved a synergistic effect combining photodynamic and photothermal antibacterial treatments. Under co-irradiation, it produced an antibacterial rate exceeding 99.1% within a short time. The hyperthermia generated by 808 nm laser irradiation weakened bacterial activity. These compromised bacteria were then readily killed through membrane disruption, protein denaturation, and destruction of bacterial metabolic pathways due to the ROS produced under 660 nm laser irradiation. This strategy of GO modification can enhance the antibacterial efficacy of CN and appears very promising for the treatment of osteomyelitis.

Due to their diverse and excellent chemical properties, metal ions are widely used in the antimicrobial field, particularly in the synthesis of MOF-based nanozymes. Metal ions can be incorporated into MOFs through various interactions. When pH changes, their coordination stability can also alter, causing the MOF structure to expose multiple metal active sites. The electrostatic interaction between cationic metals and the anionic bacterial cell wall can lead to damage of the cell wall and membrane, resulting in bacterial death (66). However, MOFs are often unstable in physiological environments, and the release of metal ions poses risks of environmental and biological toxicity. Furthermore, MOFs with rigid frameworks, often obtained as powders, are difficult to process, which significantly limits their practical application. According to the Hard-Soft Acid-Base theory, hard acids typically have small ionic radii, high oxidation states, and low polarizability. They tend to form stable metal-organic complexes when paired with hard bases, which have high electronegativity. In contrast, soft acids and borderline acids have larger ionic radii, lower oxidation states, and higher polarizability.

Consequently, they tend to form strong bonds with soft bases, such as thiol groups in proteins. The uptake of metal ions by bacteria is a critical step in their toxic action. Bacterial uptake of non-essential metals typically occurs via pathways reserved for essential organic and inorganic ions, and it is now known that transporter proteins from several families are involved in this process (67). In the MOF-based nanozyme prepared by Sun et al. (68), the presence of bimetallic components facilitated electron transfer during the catalytic process, leading to higher catalytic efficiency and enhanced catalytic and antibacterial capabilities of the Co-TCPP(Fe) MOFs. The obtained nanozyme exhibited excellent peroxidase-like activity in acidic microenvironments. Furthermore, an anti-biofilm study demonstrated that its antibacterial activity and quorum sensing disruption ability made it a remarkable inhibitor of biofilm formation (69).

### 2.2 Oxidase mimics against osteomyelitis

Naturally occurring oxidases can catalyze the oxidation of substrates into oxidized products and ROS with the assistance of molecular oxygen. These products possess remarkable antimicrobial properties. However, the practical utilization of natural oxidases is often hampered by challenges such as high cost, complex preparation and purification processes, poor long-term stability, and stringent reaction requirements. Consequently, leveraging the enzyme-mimicking functions of materials offers a pathway to achieve sustainable, environmentally friendly, and cost-effective antimicrobial outcomes. To date, oxidase mimics based on several nanomaterial systems have been reported for combating osteomyelitis (70–75).

Copper-containing nanoparticles have also been extensively explored as oxidase mimics. Although copper can effectively kill microorganisms, sublethal doses may induce a viable but non-culturable state in bacteria across many different phylogenetic lineages (76). Furthermore, one significant factor hindering the broader application of copper-based nanozymes in the antimicrobial field is their insufficient catalytic efficiency. To address this, Meng et al. (77) designed an atomically dispersed and fully exposed copper cluster, where an average of three copper atoms were stabilized on a nanodiamond-graphene hybrid support, endowing it with exceptional oxidase-like activity.

**Figure 2:**
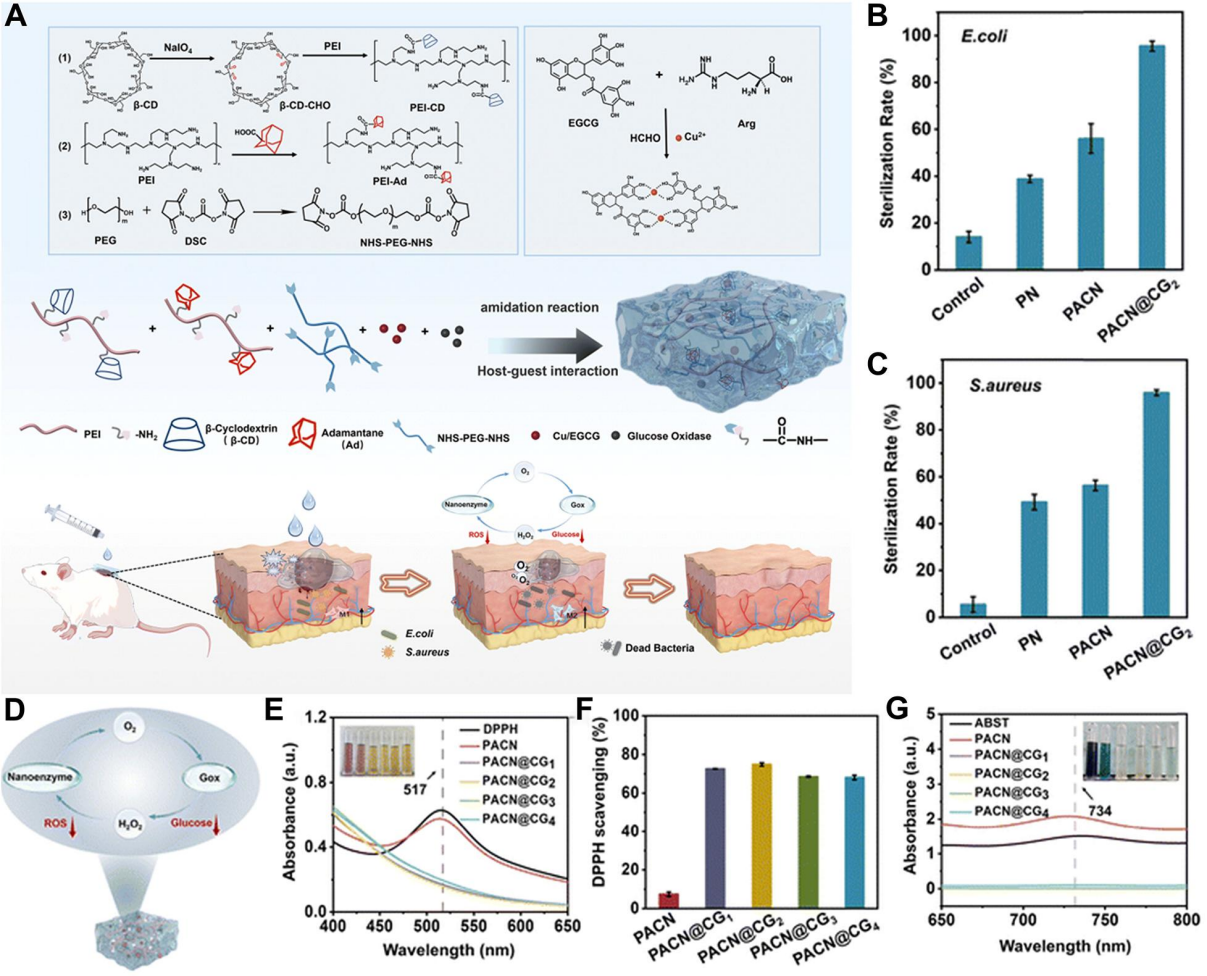
Antibacterial mechanism and effect of nanozymes (Cu/EGCG). **(A)** A dual-network supramolecular hydrogel dressing encapsulating Cu/EGCG nanozyme. Quantitative statistics of the bactericidal rates of the hydrogel against **(B)** E. coli and **(C)** S. aureus. **(D)** The cyclic mechanism under the cascade reaction. **(E)** UV absorption spectra of DPPH solutions and **(F)** quantitative analysis of DPPH scavenging ability. **(G)** UV absorption spectra of ABST solutions. All statistical data are presented as the mean ± standard deviation (SD; *n* = 3). Reproduced with permission (78).

Yu et al. developed a nanozyme composite material (Cu/EGCG nanozyme) as a multi-target combined treatment system for clinical treatment of chronic diabetic wounds (Figure 2A) (78). This system not only can alleviate hyperglycemia and combat bacterial infections, but also can relieve oxidative stress (Figures 2B–G).

The atomically dispersed and fully exposed Cu₃ clusters significantly enhance the oxidase-like activity of the nanozyme by facilitating the facile dissociation of oxygen into ROS in NaAc buffer (pH = 4.5). The generated oxygen species can intimately interact with proteins, lipids, polysaccharides, and the bacterial membrane, leading to membrane perforation and subsequent bacterial death. Plate counting experiments for the Cu₃/ND@G system against *Escherichia coli* demonstrated its potent antibacterial performance, which was superior to that of ND@G loaded with conventional copper nanoparticles (Cu-NPs/ND@G). The prepared Cu₃/ND@G, featuring atomically dispersed and fully exposed Cu₃ clusters, maximizes atomic utilization efficiency, thereby yielding exceptional oxidase-like activity. The catalytic rate constant of Cu₃/ND@G far exceeds those of reported copper-based oxidase mimics and is even approximately 15 times higher than that of commercial Pt/C mimics.

Mao et al. (72) designed and synthesized an active copper artificial oxidase based on Cu-MOFs, which catalyzes the oxidation of 3,3′,5,5′-tetramethylbenzidine (TMB) without requiring additional H₂O₂. The ligand 3-amino-5-mercapto-1,2,4-triazole (AMTA) was selected for this catalytic system due to the presence of a nitrogen and sulfur heterocyclic system, which not only confers potential bioactivity (e.g., against viral and bacterial infections) but also enables AMTA to act as a versatile ligand in coordination chemistry, strongly binding Cu^2+^ ions via N–Cu coordination to provide catalytic centers. By incorporating the triazole unit into the Cu-MOFs structure, the ROS generation efficiency was significantly enhanced. The resulting Cu-MOFs exhibited exceptional oxidase-like activity even at very low concentrations. Furthermore, this intrinsic oxidase-like activity, coupled with exogenous ROS generation, was leveraged in studies targeting bacterial strains such as *Escherichia coli* and *S. aureus*. Antibacterial results demonstrated that the Cu-MOFs possessed superior antimicrobial activity.

New-generation antimicrobial nanozymes constructed from nanoparticles (NPs) and artificial enzymes demonstrate considerable potential in the antibacterial field. Among them, gold nanoparticles (AuNPs) are particularly impressive; due to their strong synergistic coupling effects and ability to stabilize free radicals, they have been shown to effectively enhance the catalytic activity of artificial enzymes (79). Recently, researchers have successfully fabricated gold-based oxidase mimics that exhibit excellent antibacterial capability and biocompatibility. For instance, by doping AuNPs and gold iodide complexes *in situ* onto bismuth oxyhalide nanosheets, a hybrid gold/bismuth oxyhalide nanozyme with high oxidase-like activity was synthesized. The resulting Au-based nanozyme, featuring oxygen vacancies, demonstrated high catalytic activity within redox systems (80). Moreover, the Au/bismuth oxyhalide nanozyme not only displayed potent antibacterial activity against non-multidrug-resistant bacteria (such as *E. coli*, *K. pneumoniae*, *S. enteritidis*, *S. aureus*, and *B. subtilis*) but also proved highly effective against MRSA. However, the potential of Au-based enzyme mimics is still limited by their low catalytic activity at neutral pH. Developing oxidase mimics that can maintain excellent oxidase-like activity across a broad pH range—particularly within the physiological pH range—would significantly enhance their efficacy in the treatment of osteomyelitis.

Traditional graphene-based materials for antibacterial applications often require additional stimulation, such as laser irradiation for photosensitization, or the external supply of H₂O₂, which significantly compromises their ease of use and cost-effectiveness. In contrast to conventional graphene materials, the utilization of graphene quantum dot-silver nanoparticle hybrids as oxidase mimics and antibacterial agents represents a novel and promising approach (81). GQD/AgNP hybrids exhibit high oxidase-like catalytic activity and maintain good stability in neutral media across a temperature range from room temperature up to 60 °C (82). These GQD/AgNP hybrids demonstrate prominent oxidase activity, leading to excellent antibacterial performance against both Gram-negative and Gram-positive bacteria, including drug-resistant strains. They show significant potential in combating pathogens commonly associated with osteomyelitis, such as *Escherichia coli* and *S. aureus*.

### 2.3 Haloperoxidase mimics against osteomyelitis

Haloperoxidase (HPO) mimics can inhibit quorum sensing by quenching autoinducers—signaling molecules associated with this process—thereby preventing biofilm growth and promoting its disruption (83). For instance, vanadium pentoxide (V₂O₅) nanomaterials exhibiting HPO-mimetic properties have demonstrated potential as biocatalytic bactericidal agents in the presence of bromide ions (Br^−^) and H₂O₂ (84). As a result, V₂O₅, when supplemented with Br^−^ and H₂O₂, can inhibit bacterial growth more effectively than in the absence of these additives (85).

**Figure 3:**
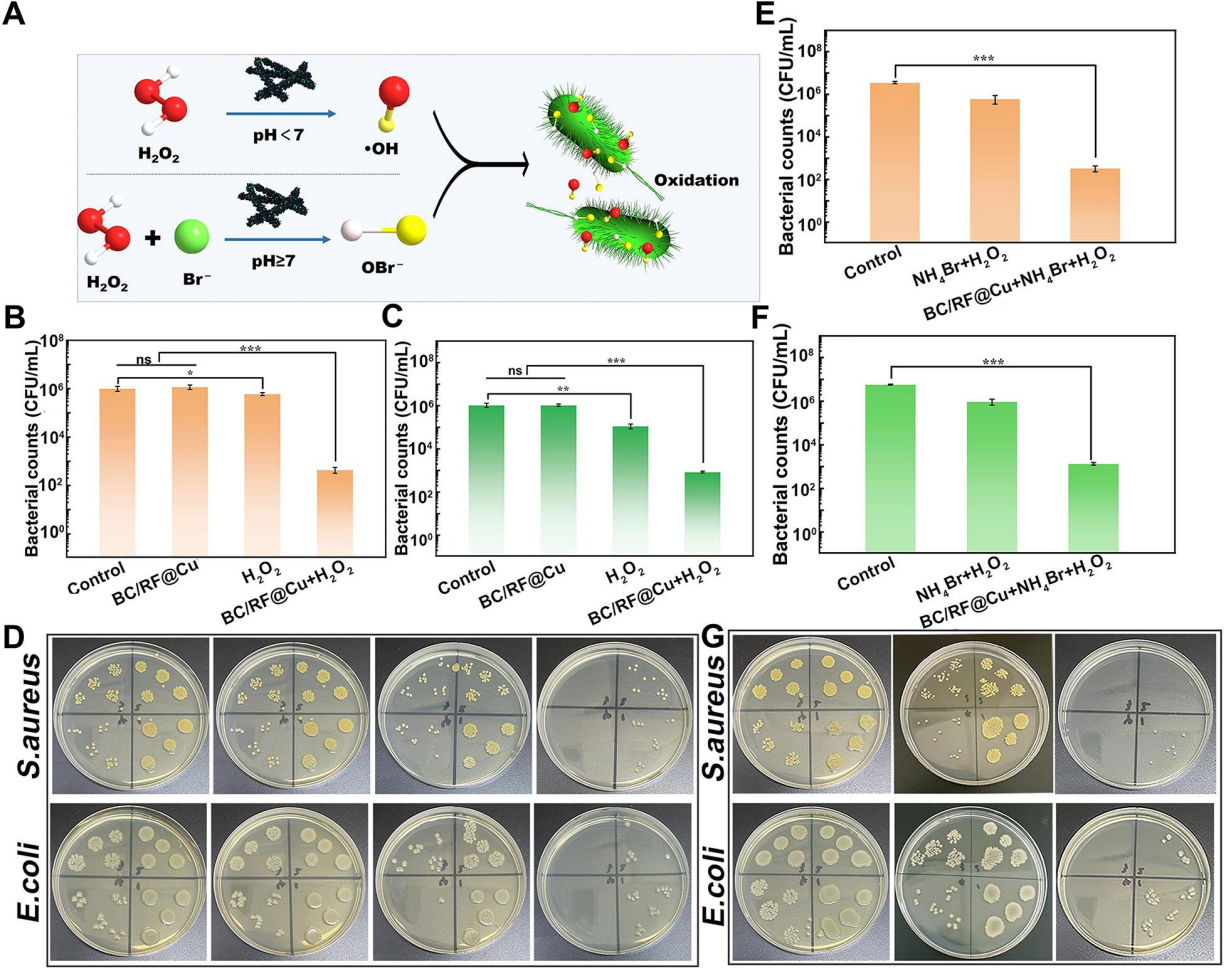
**(A)** Schematic illustration of antibacterial activity based on the catalytic performance of BC/RF@Cu. Antibacterial efficacies against **(B)** S. aureus and **(C)** E. coli based on POD-like activity, and **(D)** corresponding photographs of bacterial colonies. Antibacterial efficacies against **(E)** S. aureus and **(F)** E. coli based on HPO-like activity, and **(G)** corresponding photographs of bacterial colonies. All statistical data are presented as the mean ± standard deviation (SD; *n* = 3). Reproduced with permission (86).

Based on the enzymatic activity, the antibacterial efficacy of the ·OH and Br produced by the enzymatic activity catalyzed by BC/RF@Cu was evaluated (Figure 3A) (86). As shown in Figures 3B,C, although copper ions are potential antibacterial agents, BC/RF@Cu itself shows almost no antibacterial performance. However, the Br halogen peroxidase-like analogues exhibit good antibacterial activity (Figures 3D–G).

A significant reduction in bacterial growth was observed (78% for *E. coli* and 96% for *S. aureus*). Typically, this approach is effective for bacterial disinfection in weakly alkaline rather than acidic environments, making it suitable for applications such as marine antifouling. In another study, CeO_2−x_ nanorods were found to inhibit bacterial growth after just 5 s of incubation, owing to their morphology-dependent HPO-mimicking activity (87). More importantly, cerium-based nanocatalysts exhibit higher biocompatibility compared to vanadium-based ones, rendering them more promising for clinical antibacterial therapy (88, 89). To address the instability of natural enzymes and ensure a sufficient supply of H₂O₂, Sun et al. proposed a semiconductor-based bifunctional nanozyme for anti-biofouling (90). This nanozyme can sustainably self-supply H₂O₂ and O₂ in water through photosynthesis, thereby driving sequential HPO-mimetic reactions (91).

## 3 Mimicking hydrolases for the treatment of osteomyelitis

**Figure 4:**
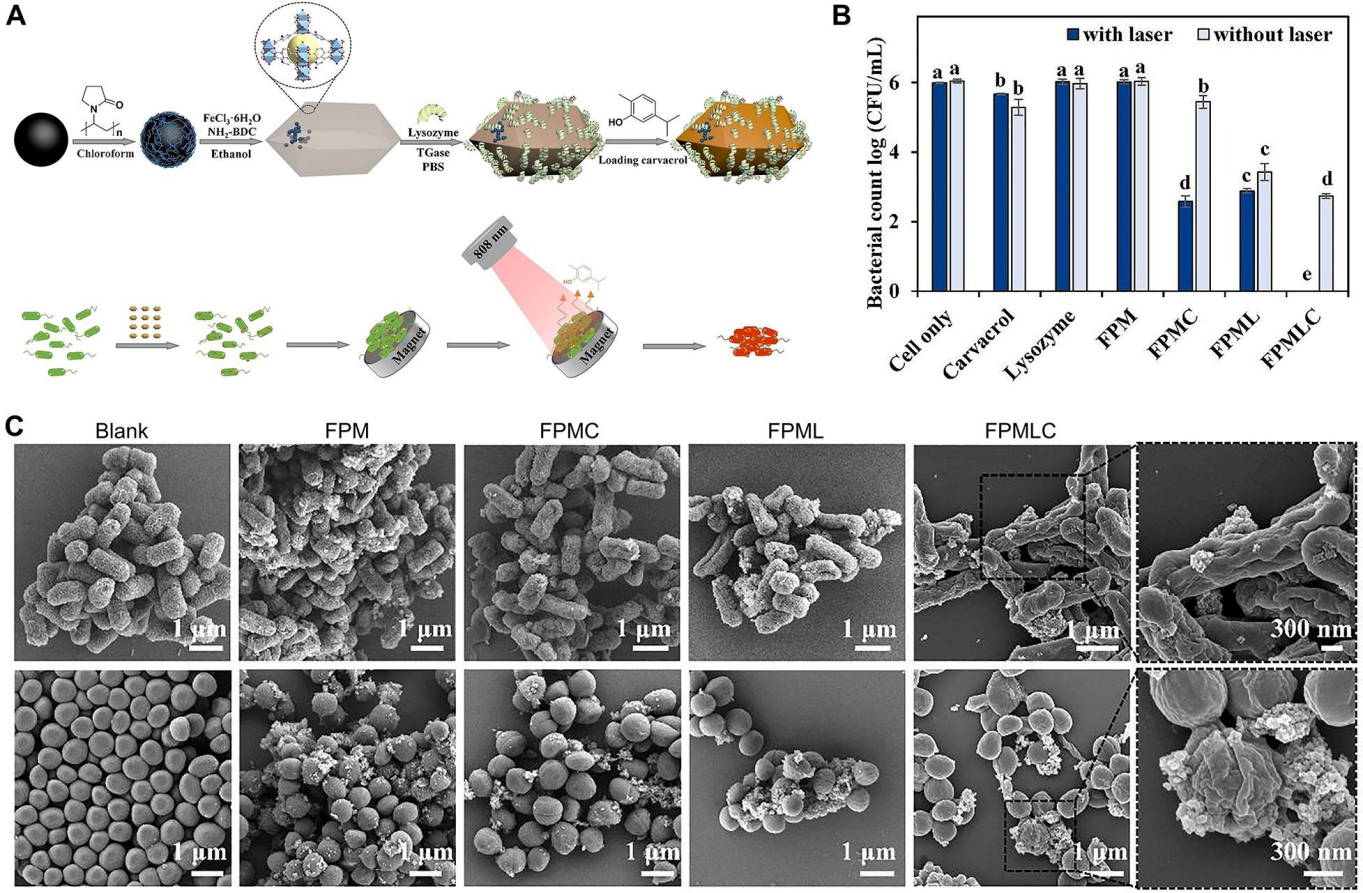
Antibacterial mechanism and effect of nanozymes (FPMLC). **(A)** Schematic diagram of the synthesis of nanozyme composite materials and the process of bacterial eradication. **(B)** Quantitative determination of bacterial count numbers of *S. aureus.* **(C)** *S. aureus* suffered different treatments. Error bars represented the standard deviation. All statistical data are presented as the mean ± standard deviation (SD; *n* = 3). Reproduced with permission (94).

Unlike oxidoreductases, which exert antibacterial effects by generating ROS to disrupt bacterial structures, hydrolases act by directly hydrolyzing essential macromolecules for bacterial viability, such as polysaccharides in the cell wall, lipids in the cell membrane, and extracellular DNA within the biofilm matrix. This physical disruption mechanism is less likely to induce bacterial resistance, offering an attractive alternative strategy against osteomyelitis (92). However, similar to oxidoreductases, the practical application of natural hydrolases is hampered by challenges such as poor stability, high cost, and sensitivity to environmental conditions. Consequently, developing nanozymes with hydrolase-like activity has emerged as an important research direction (93). Currently, nanozymes mimicking hydrolase function primarily operate by hydrolyzing key components of the bacterial cell membrane/wall or the biofilm matrix. Nong et al. developed a MOF-based nanozyme hybrid FPMLC, which was able to completely inactivate (100%) Escherichia coli and *S. aureus* at a low dose of 100 μg/mL (Figure 4) (94).

Unlike oxidoreductases that destroy bacterial structures by generating ROS, hydrolases exert antimicrobial effects by directly hydrolyzing macromolecules essential for bacterial viability, such as polysaccharides in the cell wall, lipids in the cell membrane, and extracellular DNA (eDNA) within the biofilm matrix (95). This physical disruption mechanism is less likely to induce bacterial resistance, offering a highly attractive alternative strategy for combating osteomyelitis (96). However, similar to oxidoreductases, the practical application of natural hydrolases faces challenges such as poor stability, high cost, and sensitivity to environmental conditions. Therefore, developing nanozymes with hydrolase-like activity has become an important research focus.

Currently, hydrolase-mimicking nanozymes function primarily by hydrolyzing key components of the bacterial cell membrane/wall or the biofilm matrix. The bacterial cell wall and membrane are critical structures maintaining cellular morphology and integrity (97). Nanozymes mimicking hydrolases catalyze the hydrolysis of phospholipids and glycolipids within these structures, leading to increased membrane permeability, leakage of cellular contents, and ultimately bacterial death (98).

MOF, with their tunable structures and metal active sites, show great potential for mimicking hydrolases. As mentioned in Part 2, MOF-based nanozymes can exert antibacterial effects not only by releasing metal ions or generating ROS but also because their metal nodes (e.g., Zn^2+^, Cu^2+^, Co^2+^) can act as Lewis acids, catalyzing the hydrolysis of phosphoester bonds in the bacterial cell membrane. This hydrolysis-based mechanism, being independent of substrates like H₂O₂, offers broader applicability under physiological conditions (99). For instance, zinc-containing MOFs have been shown to effectively mimic phosphatase activity, disrupting the lipopolysaccharide layer of Gram-negative bacteria (e.g., E. coli) and the peptidoglycan layer of Gram-positive bacteria (e.g., S. aureus), demonstrating broad-spectrum antibacterial activity against common pathogens of osteomyelitis.

Bacterial biofilms are a core factor contributing to the difficulty in treating osteomyelitis and its high recurrence rate. The main components of biofilms include extracellular polymeric substances (EPS), proteins, and eDNA (100). Nanozymes designed to mimic enzymes that hydrolyze these components (e.g., deoxyribonuclease DNase, polysaccharide hydrolases) can directly disrupt the physical structure of the biofilm, exposing the embedded bacteria to the immune system and antimicrobial agents, thereby enabling efficient biofilm eradication (101). Although the document explicitly states that polysaccharide hydrolases within the hydrolase system act by hydrolyzing polysaccharide components in eDNA, cell membranes, and cell walls, research on nanozymes mimicking DNase activity to target eDNA is currently more prominent (102). For example, studies designing nanomaterials with DNase-like activity (e.g., CeO₂-based nanozymes) have shown effectiveness in cleaving the eDNA network within biofilms, significantly reducing biofilm adhesion and stability (103, 104). This strategy, combined with the ability of haloperoxidase mimics (mentioned in Part 2) to quench quorum-sensing signals, can synergistically inhibit and disrupt biofilms from both physical structural and chemical signaling aspects, offering new hope for eradicating osteomyelitis (92).

Future research should focus on rational material design (e.g., tuning nanozyme size, morphology, surface charge, and chemical composition) to enhance their hydrolytic activity and selectivity (105, 106). Developing multifunctional or stimulus-responsive nanozymes and combining hydrolase mimics with other antimicrobial strategies (e.g., ROS generation, ion release) represent promising directions for creating more effective and targeted therapies for osteomyelitis.

## 4 Mimicking multi-enzyme systems for the treatment of osteomyelitis

**Figure 5:**
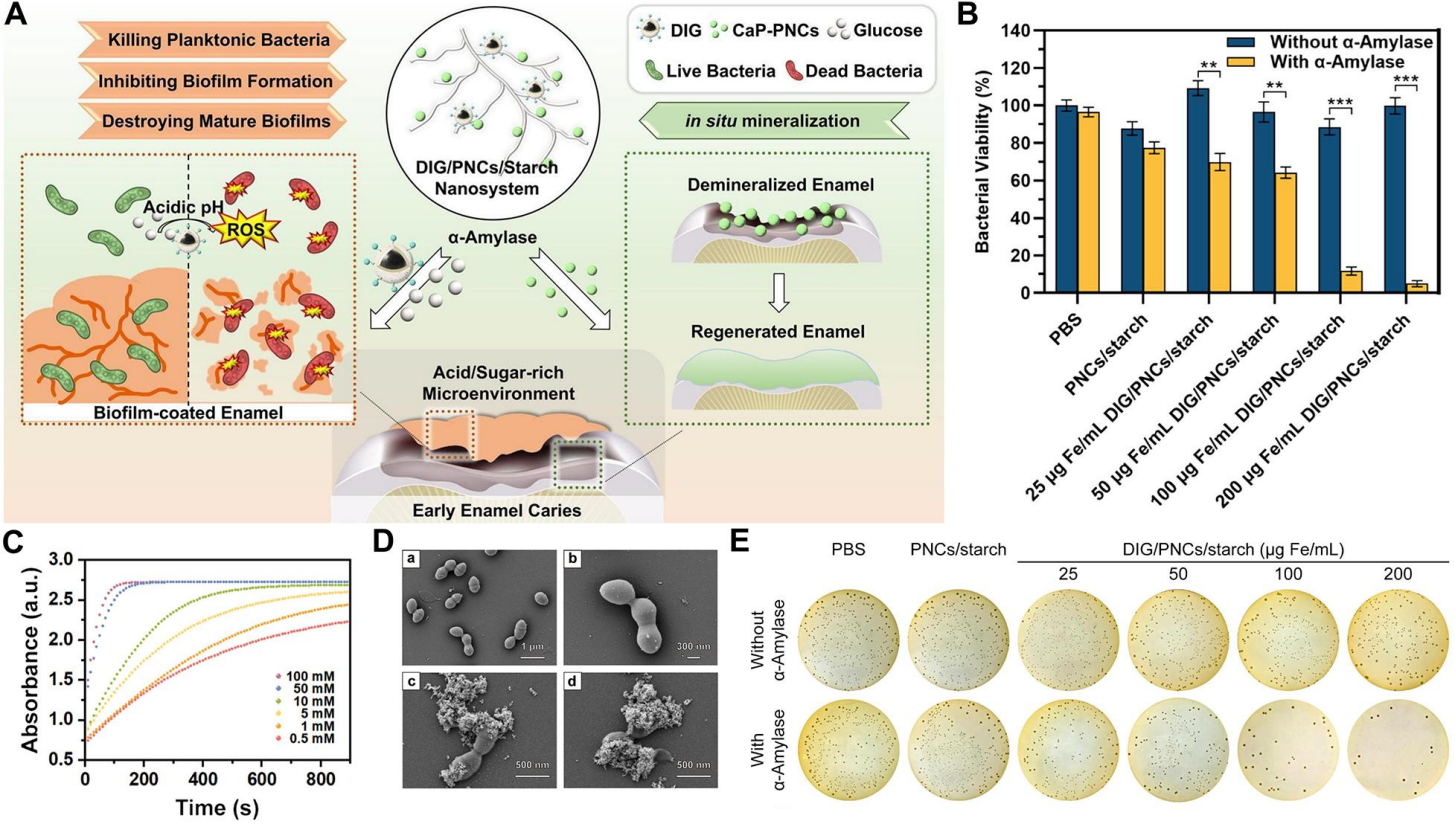
Antibacterial mechanism and effect of multiple enzyme mimics. **(A)** A schematic diagram of the mechanism of action of multiple enzyme mimics. **(B)** Bacterial count results obtained by plating counting method. **(C)** Absorbance of TMB chromogenic curves of DIG in the presence of H2O2 at different H2O2 concentrations. **(D)** SEM images of planktonic bacteria after DIG treatment. **(E)** Bacterial culture results of S. mutans treated with DIG/CaP-PNCs/starch nanosystem and *α*-amylase. All statistical data are presented as the mean ± standard deviation (SD; *n* = 3). Reproduced with permission (127).

The pathological microenvironment of osteomyelitis is complex, characterized by bacterial biofilm formation, intracellular parasitism of bacteria, and a persistent inflammatory response. A single antibacterial mechanism often proves inadequate for effectively eradicating the infection and is prone to inducing drug resistance (107). As discussed previously, nanozymes with single enzyme-mimicking activities each have advantages but also face limitations in treating osteomyelitis; for instance, peroxidase mimics depend on an acidic pH and the supply of H₂O₂, while the catalytic efficiency of hydrolase mimics may be insufficient (108). To overcome these hurdles, researchers have turned their attention to nanozyme systems capable of mimicking multiple enzymatic activities (109). These systems integrate two or more enzyme-mimicking functions on a single nanoplatform, enabling a multi-pronged, synergistic attack against bacterial infections through cooperative effects (110). This approach significantly enhances antibacterial and biofilm eradication outcomes, offering a more robust strategy for the treatment of osteomyelitis. Huang et al. developed a multi-enzyme cascade nanosystem with antibacterial properties (Figure 5A) (77). The IONP with multiple enzymatic activities can trigger the production of H2O2, generating ROS (Figures 5B,C). This process has antibacterial activity, effectively inhibiting the adhesion and biofilm formation of Streptococcus mutans (Figures 5D,E).

For instance, an ideal nanozyme might simultaneously mimic peroxidase (POD) to catalyze endogenous H₂O₂ into highly toxic •OH, and catalase (CAT) to decompose H₂O₂ into oxygen (111). This process, while seemingly contradictory, is remarkably sophisticated: the catalase-like activity alleviates hypoxia at the infection site, and the improvement of the hypoxic microenvironment not only enhances the function of immune cells but also, by increasing the metabolic activity of bacteria, renders them more susceptible to the ROS generated subsequently by the peroxidase-like activity (112). The Cu SASs/NPC nanozyme mentioned in the document, which possesses both peroxidase-like activity and the ability to deplete the bacterial self-protective molecule glutathione (GSH), essentially represents a synergy between catalytic killing and metabolic inhibition, significantly enhancing bactericidal efficiency (113).

Another powerful combination is the integration of oxidoreductase-mimicking activity with hydrolase-mimicking activity (114). This strategy allows for a concurrent “chemical attack” and “physical destruction” on bacteria. For example, a nanomaterial possessing both peroxidase-like and deoxyribonuclease (DNase)-like activities can not only oxidatively damage bacterial proteins and lipids via ROS but also directly hydrolyze extracellular DNA (eDNA) within the biofilm matrix, causing the physical collapse of the robust biofilm structure (115). This dual chemical and physical assault exposes the previously protected bacteria, leading to their effective clearance (116). MOF hold great potential in this regard, as their unstable metal-ion coordination sites can mimic hydrolase activity, while the metal nodes or the framework itself may possess redox activity, providing an ideal platform for achieving such synergy (117).

Furthermore, combining the catalytic activity of nanozymes with effects triggered by external physical stimuli, particularly photothermal therapy, is highly effective. As repeatedly noted in the document, nanozymes such as those based on gold and copper exhibit good photothermal conversion capabilities (118). Under near-infrared light irradiation, these nanozymes convert light energy into heat, generating localized hyperthermia (119). This photothermal effect itself can disrupt bacterial membrane structures and denature proteins, directly killing bacteria. More importantly, the temperature increase significantly accelerates the rates of chemical reactions, thereby enhancing the catalytic activity of nanozymes to produce more ROS, resulting in a synergistic effect between photothermal and catalytic therapies (120–122). The work by Wang et al. demonstrated that the peroxidase-like activity of Cu SASs/NPC was enhanced under NIR irradiation, achieving a synergistic photothermal-chemodynamic antibacterial therapy with nearly 100% efficiency against MRSA (123).

Despite the promising prospects of multi-enzyme-mimicking nanozymes, their design and synthesis present challenges requiring precise control. The optimal microenvironments for the catalytic centers of different enzyme-mimicking activities might differ (124). Balancing multiple activities on a single nanostructure to ensure they all function efficiently within the complex *in vivo* environment is a key research focus. Additionally, issues such as the biosafety of nanozyme systems, their ability to target and accumulate at the infection site, and the feasibility of large-scale production require further in-depth investigation (125). However, with advances in nanotechnology and synthetic biology, designing intelligently responsive multi-enzyme-mimicking nanoplatforms that can activate specific functions based on signals within the osteomyelitis microenvironment will be an important future direction (126). Such highly integrated “all-in-one” nanotherapeutic strategies hold the potential to ultimately overcome the clinical challenge of osteomyelitis.

## 5 Summary and outlook

The current clinical management of osteomyelitis primarily relies on systemic antibiotic administration. However, the escalating issue of bacterial antibiotic resistance is rendering such infections increasingly challenging to treat. This review has summarized recent advances in nanozyme-based therapeutic delivery systems for combating osteomyelitis. Various nanozymes exhibiting peroxidase-like, oxidase-like, haloperoxidase-like, and hydrolase-like activities have been developed, demonstrating strong antibacterial and anti-biofilm potential.

Compared to traditional antibiotics, nanozymes offer advantages such as high catalytic activity, good stability, and relatively low cost. Furthermore, their multi-faceted mechanisms of action—including generating ROS or physically disrupting bacterial structures—make them less prone to inducing bacterial resistance. Nanozymes can penetrate bacterial cell membranes and catalyze the conversion of endogenous H₂O₂ into highly toxic •OH, which damage bacterial lipids, proteins, and DNA. Additionally, certain nanozymes can degrade the biofilm matrix by mimicking hydrolases or induce localized hyperthermia under near-infrared light irradiation via their inherent photothermal effects. Consequently, the antibacterial efficacy of nanozymes does not rely on a single mechanism. Their application in targeting and multimodal synergistic antibacterial strategies leverages the synergistic interactions between different antimicrobial modalities, enabling enhanced efficacy at lower concentrations and reducing the emergence of resistant strains through multi-target approaches.

Despite these significant advantages, several challenges hinder the widespread adoption and clinical translation of antimicrobial nanozymes. Firstly, the intrinsic catalytic performance of nanozymes is often insufficient under physiological neutral pH conditions. Moreover, their inability to effectively distinguish between bacterial and mammalian cells may lead to damage to healthy tissues. Toxicological studies indicate that the biosafety of nanozymes is closely linked to factors such as their chemical composition, size, and surface modifications. Therefore, prior to clinical translation, standardizing preparation protocols, ensuring rigorous quality control, and conducting thorough long-term biosafety assessments are crucial steps.

The fate, degradation mechanisms, and metabolic pathways of nanozymes within the complex microenvironment of osteomyelitis infection remain poorly understood. Utilizing advanced omics technologies to study the interactions between nanozymes and the host immune system will accelerate their clinical translation. Furthermore, most current antimicrobial nanozymes are broad-spectrum agents; while eradicating pathogens, they might also disrupt beneficial microbiota. Developing intelligent nanozymes capable of specifically targeting pathogenic bacteria would significantly broaden their application prospects.

Current research on antimicrobial nanozymes predominantly focuses on single *in vitro* models or simple wound infection models. There is a clear need for validation in more complex animal models of osteomyelitis and an expansion of efficacy evaluations against deep tissue infections and chronic biofilm-associated infections. The clinical application of nanozymes is still in its infancy, lacking large-scale clinical trial data to support their efficacy and safety. Thus, enriching disease models and increasing preclinical studies are essential for advancing nanozymes toward clinical use.

With advancements in nanosynthesis techniques and characterization methods, researchers can precisely tune the structure-activity relationships of nanozymes. Concurrently, in-depth biological studies provide a solid foundation for understanding their mechanisms of action both *in vitro* and *in vivo*. It is anticipated that the application of nanozymes in the antimicrobial field, particularly for treating challenging infections like osteomyelitis, will experience rapid growth in the coming years. This review aims to assist researchers in clarifying the antibacterial mechanisms of nanozymes and provide guidance for developing novel antimicrobial nanozymes. Nanozymes with enhanced antibacterial activity and good biocompatibility hold promise for potentially becoming first-line agents against bacterial infections in the foreseeable future.
